# An anatomical study of lumbar epidural catheterization

**DOI:** 10.1186/s12871-015-0069-x

**Published:** 2015-06-23

**Authors:** Huanwei Jiang, Benchao Shi, Shiyuan Xu

**Affiliations:** 1 Department of Anesthesiology, Zhujiang Hospital, Southern Medical University, Guangzhou, China; 2 Department of Anesthesiology, Shenzhen Nanshan District Maternity & Child Healthcare Hospita, Shenzhen, China; 3 Department of Orthopedics, Zhujiang Hospital, Southern Medical University, Guangzhou, China

**Keywords:** Meningo-vertebral ligaments, Posterior epidural space, Epidural Catheterization

## Abstract

**Background:**

We herein provide an analysis of lumbar epidural catheterization, which outlines a detailed anatomical description of the epidural anatomy, and may improve the success rate of neuraxial cannulation.

**Methods:**

Lumbar epidural catheters were placed in 50 adult embalmed cadavers. After catheterization, the lumbar dura and connecting structures between the epidural space and the vertebral body were separated. The positional relationship between the catheter and the posterior epidural space were observed and photographed.

**Results:**

Amongst the 50 specimens, the epidural catheter curled into a circle in three cases, entered the intervertebral foramen in two cases, and caused epidural venous damage in five cases.

**Conclusions:**

Meningo-vertebral ligaments exist in the posterior epidural space and connect to the venous plexus, which may contribute to epidural catheter failure, uneven distribution of anaesthesia and epidural hemorrhage. Our study provides anaesthesiologists with a better understanding of the anatomy and may mitigate complications of lumbar epidural catheter placement.

## Background

Meningo-vertebral ligaments have been described between the dura mater and the lumbosacral spinal canal [1]. These structures may be responsible for intermittent failure of epidural catheterization. To explore the anatomical reasons for epidural catheterization failure, reveal the relationship between the dorsal meningo-vertebral ligaments, and thereby lower the failure occurrence rate and its related complications. This research simulated the insertion of epidural catheters in cadaveric specimens and observed the relationship between the relevant structures.

## Methods

The study was approved by Medical Ethics Committee of Southern Medical University affiliated Zhujiang Hospital, China. After obtaining informed consent from relative, fifty adult embalmed cadavers were analyzed at the Department of Anatomy, Southern Medical University, China. These included 28 male cadavers and 22 female cadavers with a median age of 55 years (range: 43–72). All cadavers had intact lumbosacral anatomy. Cadavers with known lumbosacral deformities, lumbosacral diseases or previous spine surgery were excluded from the analysis.

An 18-gauge Tuohy needle (Haining green health medical products Ind. Sdn. Bhd, China) was used and the epidural space was identified via a midline approach at the L3-L4 interspace using the loss-of-resistance-to-air technique. After the epidural space was located, a 20-gauge multiorifice catheter was threaded through the cranially directed tip of the epidural needle to the 15 cm mark. The needle was removed, and the catheter was left 6 cm into the epidural space. The spine was transected at the level of the intervertebral disc between T12 and L1. The posterior aspect of the vertebral column was exposed from L1 to S1 by dissecting the paraspinous musculature. The relationship of epidural catheter with the surrounding tissue was observed. The dura mater was then carefully reflected ventrally to expose and isolate the meningo-vertebral ligaments from the peridural membrane using blunt dissection and any extradural fat was removed. The dural sac was elevated to identify the meningo-vertebral ligaments. The distribution, morphology, number, orientation, and sites of origin and insertion of the meningo-vertebral ligaments were observed. All results were photographed and recorded.

## Results

### The relationship of epidural catheter with the surrounding tissue

**Fig. 1:**
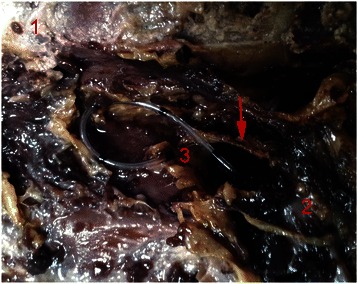
(**1**) the lamina, (**2**) the dura mater, (**3**) epidural catheter tip. The arrow points to the meningo-vertebral ligaments, which was highly distorted to “9” shape

**Fig. 2:**
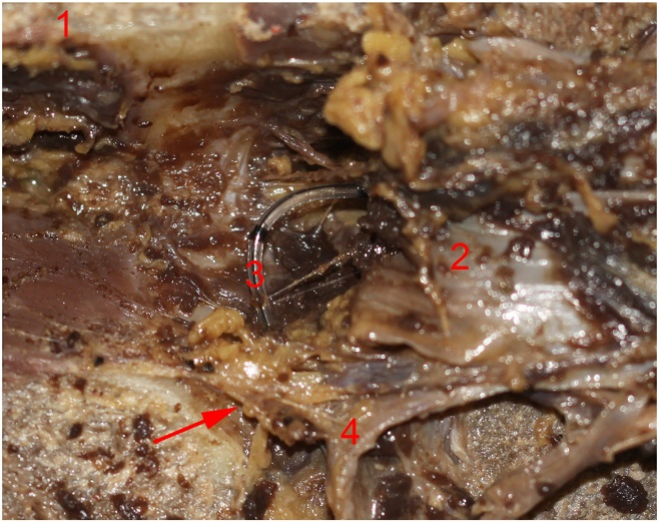
(**1**) the lamina, (**2**) the dura mater, (**3**) epidural catheter tip. (**4**) the nerve root. The arrow points to the meningo-vertebral ligaments, showing the catheter reaches the nerve root foramen

**Fig. 3:**
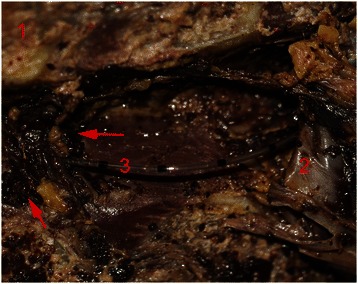
(**1**) the lamina, (**2**) the dura mater, (**3**) epidural catheter tip. The arrow is within the vertebral venous plexus, showing the catheter tip was pierced into the vertebral venous plexus

After opening the epidural space, the location of the catheter was observed and recorded. The catheter tip was attached to the meningo-vertebral ligaments and curled into a circle in three cases, with one catheter twisted into a “9” shape (Fig. 1). In two other cases, the catheter tip was in close proximity to the nerve roots near the intervertebral foramen (Fig. 2), and perforation of the vertebral venous plexus was observed in five cases (Fig. 3).

### Meningo-vertebral ligaments and their attachment sites

**Fig. 4:**
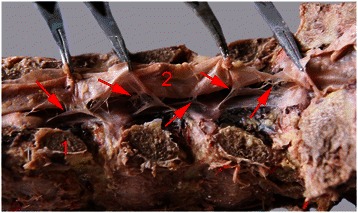
(**1**) the lamina, (**2**) the dura mater. The arrow pointed to the meningo-vertebral ligaments, the morphology of the meningo-vertebral ligaments various from the elongated bar to a large thick tough sheet, some as fine as silk, some as thick as pasta, some even forming a sagittal septum, distributed as cobweb-like

In the posterior epidural space, meningo-vertebral ligaments were observed in each of the 50 adult lumbar vertebral segments, with topographic variations between subjects and between levels. The shape of meningo-vertebral ligaments varied from a thin elongated strip to a thick tough sheet. Other meningo-vertebral ligaments formed a sagittal septum, and were oriented in a web (Fig. 4). Dural ligaments are mainly distributed in the middle or near the middle of the epidural space. The dorsal meningo-vertebral ligaments in the lumbosacral region connect the dura to the ligamentum flavum or the lamina. The dorsal meningo-vertebral ligaments were associated more often with the ligamenta flava than the lamina. Some ligaments connected directly to the spinal vascular wall. Malposition of the epidural catheter was more often observed when sheet-type and median-sagittal meningo-vertebral ligaments were present. The distribution of meningo-vertebral ligaments and the incidence of abnormal catheterization were shown in Table 1.

**Table 1:** Distribution of membrane vertebral ligament and the incidence of abnormal catheterization (%)

Vertebral Segment	Occurrence rate of dorsal meningovertebral ligaments	Incidence of catheter exit through the foramen	Incidence of catheter curled into a circle	Venous plexus puncture rate
L1	22 %(11)	0	0	0
L1-L2	32 %(16)	0	0	0
L2	40 %(20)	0	0	0
L2-L3	38 %(19)	2 %(1)	2 %(1)	2 %(1)
L3	34 %(17)	0	0	4 %(2)
L3-L4	44 %(22)	2 %(1)	4 %(2)	4 %(2)
L4	46 %(23)	0	0	0
L4-5	62 %(31)	0	0	0
L5	30 %(15)	0	0	0
L5-S1	98 %(49)	0	0	0

Values given are percentage (number)

## Discussion

It is commonly accepted that the epidural space is a continuous compartment containing fat, lymphatics, arteries, loose areolar connective tissue, spinal nerve roots, and an extensive plexus of veins [2, 3]. However, recent studies have suggested that meningo-vertebral ligaments are present between the lumbosacral spinal canal wall and the surrounding dura. Meningo-vertebral ligaments scattered in the epidural space with adipose tissue on their surface separate the epidural space into compartments of different sizes and shapes [4], which results in discontinuity of the epidural space, resulting in uneven distribution of injectate, thereby contributing to unilateral or partial anaesthesia after injection of local anaesthetic [5]. The failure rate for neuraxial labor analgesia is reported as high as 12 % [6]. When the initial epidural placement and infusion does not result in an adequate block, anesthesiologists often perform 2 different maneuvers to salvage the catheter: partial withdrawal of the catheter or infusion of additional medication [7]. If all these 2 maneuvers couldn’t lead to a successful analgesia, anaesthetist should try other maneuvers such as replacement of the epidural catheter or a combined spinal epidural anaesthesia.

Meningo-vertebral ligaments located within the epidural space may contribute to epidural catheter placement failure [8]. The location of these structures can be unpredictable with 38 % of meningo-vertebral ligaments located in the middle of the subdural cavity, 32 % in the left side subdural cavity, and another 30 % next to right side of subdural cavity [9]. Our study found three epidural catheters curled into a circle in 3 cases, with one catheter curled into the shape of a “9”. We hypothesise that these structures may contribute to catheter knotting within the epidural space [10]. The conclusion of some reports is that insertion of excessive amounts of catheter into the epidural space is a causative factor in knot formation [11]. Some authors have recommended the insertion of no more than 4 cm of catheter into the epidural space and some others no more than 5 cm [12, 13]. According to the study of Aslanidis T et al. [14], the 6 cm of catheter left into the epidural space in the current case was not excessive. This may not become apparent until removal of the catheter, at which time, catheter fracture may occur. Irregardless, when removing an epidural catheter, one should hesitate when resistance is encountered, change position and then slowly attempt to remove the catheter again. If necessary, surgical assistance should be sought [15, 16].

Epidural catheter was placed at the L3-L4 interspace. As it was seen at Table 1, the highest frequency of catheter curling occured at the same level. Bridenbaugh et al. reported that 48 % of the catheters coiled at the insertion site [17]. One possible reason is that a large number of meningo-vertebral ligaments at the L3-L4 interspace blocked the catheter tip. At the same time, an abrupt change of direction of the catheter tip in the epidural needle tip make it difficult to insert the catheter. Maybe, all the two reasons resulted in a higher incidence of catheter curling at the L3-L4 interspace.

Others have reported that 5 % of epidural catheters travel caudally or exit the intervertebral foramen [18]. In our study, there were two cases in which the epidural catheter traveled into the intervertebral foramen, and we hypothesise that this course may have been due to variant meningo-vertebral ligaments. In such cases, the epidural injectate would course laterally into the intervertebral foramen resulting in nerve root rather than epidural spread of the injectate.

In this study, we observed five cases where the catheter punctured the internal vertebral venous plexus. Unintended venous injury may occur during lumbar epidural catheter placement, with an estimated incidence rate of 5–7 % [6]. Yaakov berlin et al. found that patients whose catheters were threaded 7 cm into the epidural space had the highest incidence (17.5 %) of intravenous catheter placement as compared to patients whose catheters were threaded 3 or 5 cm [18]. They speculated that more catheter was threaded into the epidural space the greater was the likelihood of encountering a vein. If not recognized, local anaesthetics will enter into the systemic circulation, potentially resulting in seizure, cardiovascular toxicity, circulatory failure or death [19, 20].

Our study found that the meningo-vertebral ligaments directly connected to the wall of the spinal microvasculature in the location of lamina and ligamentum flavum. These structures were entwined with, but not adherent to the vascular walls. Earlier studies have reported that small blood vessels were found in fetal tissue specimens of meningo-vertebral ligaments [21, 22]. When performing epidural catheter placement, if the needle inadvertently damages blood vessels connected to this ligament, the risk of developing an epidural hematoma is hypothesised to increase. Vascular structures connecting to the meningo-vertebral ligaments are mostly venous, these veins lack valves and exist in a space where compression can result in catastrophic outcomes.

No inadvertent dural puncture was reported in our study. The incidence of inadvertent dural puncture ranges between 0.19-0.5 % of epidural catheter placements. Adverse events may result from direct mechanical injury or adverse physiological responses. Inadvertent dural puncture and postdural puncture headache, direct neural injury, total spinal anesthesia, and subdural block have been commonly reported.

Meningo-vertebral ligaments become thicker as they occur more caudally, with the thickest ligament at L5-S1. At the caudal levels, the meningo-vertebral ligaments are firmly connected with the ligamentum flavum, which may be the reason that intraoperative dural tearing occurs more commonly during surgery at the L4-L5 and L5-S1 levels [1].

The major limitation of this study is the lack of clinical data from live patients. Preserved cadaveric specimens, while the only suitable study population for the research which we undertook, are limited by the changes in the tissue structure with death and fixation of the body with formalin. It is well known that tissue fixation and its associated desiccation can lead to tissue shrinkage. The use of formalin may have contributed to an overestimation of the strength of the ligaments than they are in vivo. Maybe, it is one of the reasons why the incidence (10 %) of intravenous catheter placement in our study was much lower than that (17.5 %) in vivo.

## Conclusions

 In short, meningo-vertebral ligaments are present in lumbosacral epidural retrodiscal space with various shapes. They divide the epidural space into compartments of various sizes, which are sometimes connected with the venous plexus and can be implicated as a cause of epidural catheter failure, uneven distribution of anaesthesia and epidural hemorrhage. Anaesthesiologists should be aware of the existence of these ligaments, and their potential role in the failure of epidural anaesthesia.
